# The Association Between Inclusive Leadership and Job Performance in Nurses: Exploring the Mediation Roles of Grit and Work Engagement Based on a Cross‐Sectional Study

**DOI:** 10.1155/jonm/9399937

**Published:** 2026-06-30

**Authors:** Yue Wen, Jing Liao, Chunyan Lu, Lan Huang, Yanling Ma, Wentao Bo, Song Wang

**Affiliations:** ^1^ Department of Gastrointestinal Surgery, West China Hospital, Sichuan University/West China School of Nursing, Sichuan University, Chengdu, China, scu.edu.cn; ^2^ Department of Hepatopancreatobiliary Surgery, Sichuan Clinical Research Center for Cancer, Sichuan Cancer Hospital and Institute, Affiliated Cancer Hospital of University of Electronic Science and Technology of China, Chengdu, China; ^3^ Department of Radiology, West China Hospital, Sichuan University, Chengdu, China, scu.edu.cn

**Keywords:** grit, inclusive leadership, job performance, nurses, work engagement

## Abstract

**Background:**

The demanding nature of nursing makes job performance critical for patient safety. While inclusive leadership is recognized for its positive association with nurse outcomes, the mechanisms linking it to job performance remain underexplored. This study examines whether grit and work engagement statistically mediate this link.

**Methods:**

A cross‐sectional survey was conducted using a convenient sampling method among 429 registered nurses in Sichuan Province, China, between July and August 2025. Participants completed validated measures assessing inclusive leadership, grit, work engagement, and job performance. Statistical analyses, including descriptive statistics, correlations, and common method bias checks, were performed using IBM SPSS 26.0. Multiple mediation analyses were subsequently conducted using the SPSS PROCESS macro.

**Results:**

Inclusive leadership was positively correlated with grit, work engagement, and job performance (all *p* < 0.001). The mediation analysis revealed that the total effect of inclusive leadership on job performance was significant (*β* = 0.29, *p* < 0.001). However, the direct effect of inclusive leadership on job performance became nonsignificant (*β* = 0.04, *p* > 0.05) when the mediators were included. Significant indirect effects were found via grit alone (*β* = 0.12, *p* < 0.001), work engagement alone (*β* = 0.11, *p* < 0.001), and sequentially via grit followed by work engagement (*β* = 0.02, *p* < 0.001).

**Conclusion:**

Inclusive leadership is positively associated with nurses’ job performance, and this link is statistically mediated by grit and work engagement, both individually and sequentially. Healthcare organizations should invest in leadership training that fosters openness and accessibility, alongside programs building nurses’ grit and work engagement, to enhance their job performance and potentially improve patient outcomes indirectly.

## 1. Introduction

The nursing profession is the backbone of healthcare delivery, a demanding field characterized by high‐stakes environments, emotional labor, and relentless pressure [1]. Within this context, nurses’ job performance—defined as the behaviors and activities nurses are expected to undertake as part of their formal job responsibilities, including clinical tasks, patient care coordination, documentation, and adherence to safety protocols [2]—is widely recognized as having an important relationship with patient safety, quality of care, and overall organizational efficacy [3]. However, healthcare systems worldwide are grappling with a crisis of nurse burnout, turnover, and staffing shortages, all of which threaten to undermine this crucial performance [4]. Consequently, identifying factors that can enhance and sustain nurse job performance has become a paramount concern for healthcare researchers and administrators alike.

A substantial body of evidence indicates that leadership style is one of the most potent organizational factors influencing nurse outcomes. Transformational, authentic, and ethical leadership styles have been extensively studied and linked to improved nurse performance, job satisfaction, and well‐being [5]. In recent years, however, the concept of inclusive leadership has emerged as particularly salient for the modern, diverse, and interprofessional healthcare environment [6]. Unlike transformational leadership, which highlights vision and inspiration [7], inclusive leadership specifically focuses on actively inviting and valuing contributions from all team members regardless of their status or background [8]. Similarly, unlike authentic leadership, which emphasizes leader self‐awareness and transparency [9], inclusive leadership prioritizes leader openness to others’ input and accessibility [8]. In the nursing context—where teams increasingly include diverse generational cohorts, cultural backgrounds, and educational levels [10]—inclusive leadership may be uniquely suited to foster psychological safety and a sense of belonging among all staff members. Furthermore, in high‐stress environments like hospitals, where hierarchical dynamics can inhibit junior nurses from speaking up about patient safety concerns [11], inclusive leadership’s emphasis on availability and openness may prove particularly critical.

Rooted in the principles of openness, accessibility, and availability, inclusive leadership is defined as a leadership style that actively invites and appreciates contributions from all team members, fosters a climate of psychological safety, and empowers individuals by recognizing their unique value [8]. Existing studies suggest that inclusive leadership positively correlates with various aspects of nurses’ work life, including job performance, innovative behavior, and well‐being [12–15]. When nurses perceive their leaders as inclusive, they are more likely to feel a sense of belonging and connection with their organization, which is associated with increased motivation and efficacy [16]. While these findings are encouraging, the mechanisms by which inclusive leadership is linked to nurses’ job performance remain relatively underexplored. Based on job demands–resources (JD–R) theory [17] and self‐determination theory [18], we propose that the link between inclusive leadership and job performance is mediated by personal psychological resources that are essential for thriving in the demanding nursing profession.

Among these crucial psychological resources, grit and work engagement stand out as particularly relevant. Grit, defined as the trait‐level combination of passion and perseverance for long‐term goals, entails maintaining effort and interest over years despite failure, adversity, and plateaus in progress [19]. Nurses with high levels of grit are more likely to persist in the face of adversity, learn from their mistakes, and remain engaged in their work, even when faced with stressful situations or heavy workloads [20]. This unwavering dedication is associated with better job performance, as these nurses are more likely to go the extra mile for their patients and colleagues, proactively solve problems, and seek opportunities for professional development [21, 22]. Work engagement, on the other hand, refers to a positive, fulfilling, work‐related state of mind characterized by vigor, dedication, and absorption [23]. It is a well‐established correlate of job performance across various professions, including nursing [24]. Engaged nurses are more likely to be proactive, creative, and dedicated to their work, leading to enhanced performance and improved patient outcomes [25]. Schaufeli et al. argue that work engagement is not merely the absence of burnout but rather a distinct and positive psychological state characterized by energy, involvement, and efficacy [23]. These nurses are not only more productive but also more resilient to stress, which is linked to increased job satisfaction and a reduced risk of burnout [26, 27].

Based on JD–R theory [17] and self‐determination theory [18], we propose that inclusive leadership acts as a vital job resource associated with both grit and work engagement in nurses. These, in turn, are linked to improved job performance. The JD–R model posits that employees’ well‐being and performance are influenced by the balance between job demands and job resources [17]. Inclusive leadership, by providing support, recognition, and opportunities for growth, can buffer against the negative effects of job demands and promote a more positive work experience for nurses [28]. Specifically, an inclusive leader who exhibits openness, accessibility, and availability can create a supportive environment where nurses feel comfortable taking risks, sharing ideas, and seeking help when needed. This sense of psychological safety may foster a greater sense of ownership and dedication to their work, thus increasing grit [29]. From a self‐determination theory perspective, inclusive leadership satisfies nurses’ basic psychological needs for autonomy (by valuing their input), competence (by providing growth opportunities), and relatedness (by fostering belonging), thereby enhancing intrinsic motivation and work engagement [30]. Overall, when nurses feel valued and respected by their leaders, they are more likely to be energized, dedicated, and absorbed in their work, linking to improved performance.

Despite evidence linking inclusive leadership to grit, work engagement, and job performance [12–14, 29, 31], existing literature has yet to examine the sequential mediation of grit and work engagement in this context. Therefore, we propose this sequential pathway as a theoretically plausible mechanism requiring empirical testing. The proposed ordering—grit preceding work engagement—is theoretically grounded in the distinction between trait‐like and state‐like psychological constructs. Grit is conceptualized as a stable, trait‐level characteristic reflecting long‐term passion and perseverance [20], whereas work engagement is a more dynamic, state‐like psychological state that fluctuates in response to daily work conditions [23]. This temporal ordering aligns with self‐determination theory [18], which suggests that stable individual differences, such as grit, shape how individuals experience and respond to their work environment. Specifically, nurses who have developed greater perseverance (grit) are more likely to invest sustained effort in their work, which in turn generates the positive affective experiences—vigor, dedication, and absorption—that characterize work engagement [25]. In essence, grit provides the “long‐term fuel” that enables nurses to maintain engagement even when facing daily challenges. This sequential logic is also supported by empirical research showing that grit predicts subsequent work engagement over time [32, 33]. Thus, inclusive leadership may first be associated with higher grit, which then corresponds to higher work engagement, ultimately relating to improved job performance.

### 1.1. The Present Study

In general, while inclusive leadership is recognized as having a positive association with nursing outcomes, the specific mechanisms linking it to job performance remain underexplored. Although grit and work engagement have been separately linked to job performance [22, 24], and inclusive leadership has been linked with each individually [29, 31], no study to our knowledge has examined whether grit and work engagement sequentially mediate the inclusive leadership–job performance relationship in nursing populations. This sequential model is theoretically important because it distinguishes between the enduring trait of perseverance (grit) and the fluctuating state of energetic involvement (work engagement), offering a more complete and nuanced understanding of how leadership is linked to performance through multiple psychological pathways. Therefore, this study aims to investigate the link between inclusive leadership and job performance in nurses and the mediating roles of grit and work engagement in this association. Based on the existing literature and theoretical reasoning, we propose the following hypotheses (H):

 H1: There is increasing evidence of positive associations between inclusive leadership and nursing outcomes, including job performance [12–15]. Thus, inclusive leadership will be positively correlated with nurses’ job performance.
 H2: Inclusive leadership may foster grit by creating a supportive environment [28, 29], and grit has been shown to predict nurses’ job performance [21, 22]. Therefore, grit will mediate the inclusive leadership–job performance link.
 H3: Previous studies have suggested that work engagement is positively associated with inclusive leadership [14, 31] and enhanced job performance [24]. Hence, work engagement will mediate the inclusive leadership–job performance link.
 H4: According to self‐determination theory [18], individuals with a high level of grit may invest sustained effort in their work, resulting in positive emotional experiences of engagement [25]. Research has shown that grit predicts subsequent work engagement [32, 33]. Consequently, grit and work engagement will sequentially mediate the inclusive leadership–job performance link.

## 2. Methods

### 2.1. Research Design

Following a cross‐sectional, quantitative survey design, this research adhered to the Strengthening the Reporting of Observational Studies in Epidemiology (STROBE) guidelines [34] to ensure rigorous and transparent reporting of the study’s findings.

### 2.2. Participants and Procedure

A cross‐sectional survey was conducted among registered nurses from several secondary and tertiary public hospitals in Sichuan Province, China. We included both hospital levels to enhance external validity and generalizability to the broader nursing workforce in China, as healthcare systems in many regions include a mix of hospital levels with varying resources and patient acuity. Data were collected between July and August 2025 using a convenient sampling method. Sichuan Province was selected because it is one of China’s most populous provinces with a diverse healthcare system spanning urban tertiary hospitals and rural secondary facilities. This provided a reasonably representative sample of the broader Chinese nursing workforce while maintaining logistical feasibility. The survey employed an anonymous online questionnaire distributed by trained investigators. These investigators, based in their respective hospitals, provided standardized instructions to participants regarding the study’s purpose and significance. Prior to their participation, nurses were assured of anonymity and voluntary involvement, and informed consent was obtained.

Eligibility criteria included the following: (a) holding a valid nursing qualification in China; (b) a minimum of 1 year of clinical nursing experience; (c) having worked under the same direct nurse manager for at least 3 months prior to data collection (to ensure stable perceptions of leadership style); and (d) the ability to complete an online questionnaire. Nurses in clinical training programs or student nurses were excluded. Participants were instructed to rate the leadership style of their immediate nurse manager as the referent for inclusive leadership. To ensure the stability of leadership perceptions, we excluded nurses whose unit had experienced a change in nurse manager within the 3 months prior to data collection. This information was collected via a screening question at the beginning of the survey: “Has your current direct nurse manager been in position for at least 3 months?” Nurses who responded “no” were directed to the end of the survey. A total of 448 nurses were invited to participate, and 429 valid responses were received, resulting in a response rate of 95.8%.

The 19 excluded responses were omitted due to incompleteness, a lack of effort, or inconsistent response patterns (e.g., selecting the same option across all items). Departmental affiliation was not systematically recorded in this study due to concerns about participant anonymity. We acknowledge that departmental context (e.g., unit acuity, patient flow, and team composition) may influence the relationships among study variables, but we could not collect this information because some departments (e.g., intensive care units and emergency departments) have small numbers of nurses, and collecting such data could potentially allow for the identification of individual participants. The target sample size was determined a priori using G‐Power 3.1 software [35]. The following parameters were specified: a medium effect size (*f* ^2^ = 0.15, conventional for behavioral research), *α* = 0.05, power (1 − *β*) = 0.95, and three predictors in the full mediation model (inclusive leadership, grit, and work engagement). These parameters yielded a minimum required sample size of 207 participants. To account for potential incomplete surveys, lost responses, or exclusion criteria, we added a 20% buffer, resulting in a target of approximately 260 participants. Ultimately, we invited 448 nurses and obtained 429 valid responses, substantially exceeding the minimum requirement and providing robust statistical power for detecting smaller effects and conducting bootstrapping analyses [22, 35].

### 2.3. Variables and Measures

#### 2.3.1. Demographic Questionnaire

A general data questionnaire was used to collect sociodemographic information, including age, gender, marital status, education level, professional title, income (CNY/year), and length of nursing work (years). Both educational level (categorical) and years of education (continuous) were collected to allow flexibility in statistical modeling. Years of education provides a more precise continuous measure for use as a covariate in regression analyses, whereas educational level is more easily interpretable for descriptive reporting. Income was collected as a continuous variable (annual income in CNY) rather than as categorical ranges because continuous measurement preserves statistical power and allows for more precise covariate adjustment in regression analyses.

#### 2.3.2. Inclusive Leadership

Participants’ perceptions of inclusive leadership were assessed using the Inclusive Leadership Scale (ILS) [8]. This scale consists of nine items distributed across three dimensions: openness, accessibility, and availability. Responses were collected using a five‐point Likert scale, where 1 represented “*strongly disagree*” and 5 represented “*strongly agree*.” A higher composite score on the scale signifies a higher level of inclusive leadership. The ILS has proven effective in the Chinese context, establishing it as a reliable and valid instrument for assessing inclusive leadership in different populations, including nurses [13, 14]. The scale demonstrated strong internal consistency in this study, with a Cronbach’s alpha of 0.92.

#### 2.3.3. Grit

Participants’ grit levels were assessed using the 8‐item Short Grit Scale (SGS) [36, 37]. This scale, which is the Chinese version validated by Li et al. [36] and originally compiled by Duckworth & Quinn [37], consists of two dimensions: interest and effort. Participants responded on a 5‐point Likert scale, ranging from 1 (“*not at all like me*”) to 5 (“*very much like me*”). After reverse‐scoring the four items on the interest dimension, the total grit score was calculated by summing all eight responses, with higher scores indicating greater levels of grit [36, 37]. The SGS has established good reliability and validity in Chinese populations, including within nursing contexts [36, 38]. In our study, Cronbach’s alpha for the SGS was 0.72, demonstrating adequate internal reliability.

#### 2.3.4. Work Engagement

Participants’ work engagement was assessed using the Utrecht Work Engagement Scale (UWES) [23]. This instrument comprises 17 items measuring three dimensions: vigor, dedication, and absorption. Responses were captured using a 7‐point Likert scale, with anchors ranging from 1 (“*never*”) to 7 (“*always*”). Higher scores on the scale reflect a greater level of work engagement. The Chinese UWES has undergone validation and is recognized for its psychometric soundness, demonstrating reliability and validity across different populations, including nurses [39, 40]. In our study, the UWES demonstrated strong internal consistency, with a Cronbach’s alpha of 0.94.

#### 2.3.5. Job Performance

Participants’ in‐role job performance was assessed using the Task Performance Scale (TPS) [2]. We selected this unidimensional tool because it focuses specifically on core task performance—the completion of prescribed duties essential to the nursing role—rather than contextual performance (e.g., organizational citizenship behaviors) [2]. Although nursing job performance is multidimensional, encompassing clinical skills, communication, teamwork, and patient education, the TPS provides a focused assessment of how well nurses fulfill their formally assigned responsibilities. This focus aligns with our research question examining the link between leadership and basic job performance. However, we acknowledge that future research may benefit from more comprehensive measures that capture the full breadth of nursing performance, including contextual and adaptive performance dimensions. The scale has five items, for which participants indicated their agreement on a 7‐point Likert scale (1 = “*strongly disagree*” to 7 = “*strongly agree*”). The third item was reverse‐scored, and then all item scores were summed to obtain a total job performance score, with higher scores reflecting higher performance levels. The TPS has shown satisfactory reliability and validity in Chinese contexts, including for nursing populations [22, 41]. In our study, Cronbach’s alpha for the TPS was 0.75, indicating adequate internal reliability.

### 2.4. Data Analyses

Data were analyzed using IBM SPSS Statistics Version 26.0. Recognizing the potential for common method bias in self‐report data [42], Harman’s single‐factor test was initially conducted. This procedure involved performing an exploratory factor analysis on all items from the study measures. The criterion for acceptable common method bias was that a single dominant factor should explain less than 40% of the total variance [42]. Following this check, descriptive statistics and Pearson correlation analyses were computed for all study variables.

Subsequent analyses were performed using IBM SPSS 26.0 with the Hayes’ PROCESS macro [43]. Model 6 was employed to test the hypothesized mediation model, examining the link between inclusive leadership and job performance, with grit and work engagement as mediators. This approach allowed for the assessment of both the direct effect of inclusive leadership on job performance and the indirect effects mediated by grit and work engagement. The statistical significance of the mediation effects was determined using bootstrapped 95% confidence intervals (5000 iterations); exclusion of zero from the interval indicated a significant mediation effect. To assess the robustness of the findings, a secondary mediation model was developed, incorporating sociodemographic variables (age, sex, educational years, income, and length of nursing work) as covariates. In addition, to address the potential distortion caused by mixing 5‐point and 7‐point Likert scales, all variables were standardized (z‐scored) prior to mediation analyses, transforming different scale metrics into a common metric. The survey also employed interleaved item ordering across scales and included three instructed‐response attention checks to identify inattentive respondents.

### 2.5. Ethical Considerations

Ethical approval for this research was obtained from the Ethics Committee of West China Hospital of Sichuan University (approval number: 2021–1216). All participants were informed of the study’s nature, potential advantages, possible disadvantages, and the intended application of the findings before data collection commenced. Written informed consent was secured from each participant, signifying their comprehension of the study’s protocols and their voluntary and anonymous engagement.

## 3. Results

### 3.1. Demographics and Participant Characteristics

Table 1 lists the demographic characteristics of the study participants. The majority of participants were female (93.7%), and the mean age was 34.5 years (standard deviation = 8.1, range: 19–60). Most participants held a bachelor’s degree (78.3%) and were married (67.8%). The mean length of nursing work was 12.7 years (standard deviation = 8.6, range: 1–41), and the mean income was 84,500 CNY/year (standard deviation = 36,900, range: 20,000–260,000).

**TABLE 1:** Sociodemographic characteristics of the participants.

Variable	Mean ± SD (range) *N*%
Sex	
Female	402 (93.7%)
Male	27 (6.3%)
Age (years)	34.5 ± 8.1 (19–60)
Educational level	
Graduate degree	5 (1.2%)
Bachelor degree	336 (78.3%)
College degree	84 (19.6%)
Secondary vocational degree	4 (0.9%)
Marital status	
Married	291 (67.8%)
Unmarried	118 (27.5%)
Divorced	15 (3.5%)
Widowed	3 (0.7%)
Other	2 (0.5%)
Professional title	
Senior	4 (0.9%)
Vice senior	32 (7.5%)
Intermediate	202 (47.1%)
Primary	183 (42.6%)
None	8 (1.9%)
Years of education	15.8 ± 0.6 (12–19)
Income (CNY/year)	84,500 ± 36,900 (20,000–260,000)
Length of nursing work (years)	12.7 ± 8.6 (1–41)

Abbreviations: N, number; SD, standard deviation.

### 3.2. Common Method Bias Testing

Exploratory factor analysis revealed that no single factor explained the majority of the variance. The first factor explained only 17.01% of the variance, which is well below the established threshold of 40%. These results suggest that common method bias is not a significant concern in this study [42]. However, we acknowledge that this test alone does not fully rule out common method variance (see Limitations).

### 3.3. Correlations Between Study Variables

Table 2 presents the descriptive statistics and bivariate correlations for study variables. The assumption of normality was assessed by examining the skewness and kurtosis values of the study variables, which ranged from −0.86 to 0.97 (Table 1). Adhering to the criterion that absolute skewness and kurtosis values below 2 indicate a normal distribution for sample sizes larger than 300 [44], all variables met this requirement. Consequently, parametric statistics (mean ± standard deviation) were reported as descriptive statistics, and Pearson correlations were used for bivariate analyses. As hypothesized, correlation analyses revealed significant positive associations among the key variables. Inclusive leadership was positively correlated with grit (*r* = 0.36, *p* < 0.001), work engagement (*r* = 0.47, *p* < 0.001), and job performance (*r* = 0.29, *p* < 0.001). Grit was also positively correlated with work engagement (*r* = 0.35, *p* < 0.001) and job performance (*r* = 0.45, *p* < 0.001). Finally, work engagement was positively correlated with job performance (*r* = 0.41, *p* < 0.001).

**TABLE 2:** Descriptive statistics and bivariate correlations of study variables.

Variable	Mean ± SD	Range	Skewness	Kurtosis	1	2	3	4
1. Inclusive leadership	38.98 ± 6.00	12–45	−0.78	0.55	—			
2. Grit	27.63 ± 4.44	18–40	0.97	0.48	0.36	—		
3. Work engagement	85.68 ± 20.08	23–119	0.02	−0.58	0.47	0.35	—	
4. Job performance	28.86 ± 4.49	18–35	−0.32	−0.86	0.29	0.45	0.41	—

*Note:* All correlation coefficients were statistically significant at the *p* < 0.001 level.

Abbreviation: SD, standard deviation.

### 3.4. Mediation Results

**FIGURE 1:**
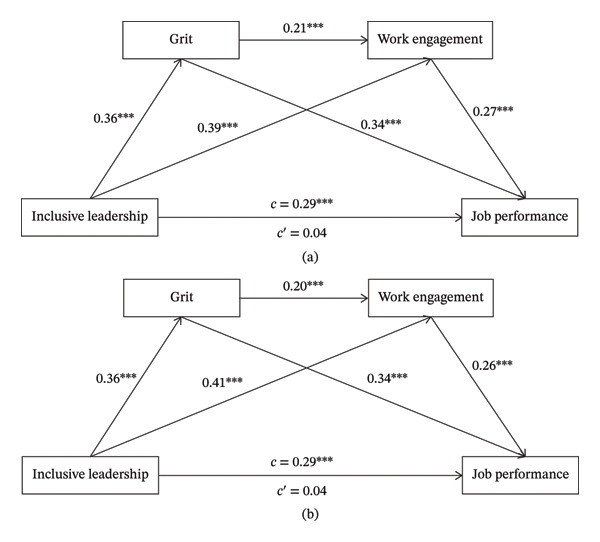
Model of the mediation role of grit and work engagement in the relation between inclusive leadership and job performance. (a) No covariates were controlled for in the model. (b) Sociodemographic variables (age, sex, years of education, income, and length of nursing work) were controlled for in the model. Standardized regression coefficients were displayed in the path diagram; c, total effect; c′, direct effect. ^∗∗∗^, *p* < 0.001.

Table 3 and Figure 1a present the results of the mediation analysis without covariates. The total effect of inclusive leadership on job performance was significant (*β* = 0.29, *p* < 0.001). After accounting for the mediators of grit and work engagement, the direct effect of inclusive leadership on job performance became nonsignificant (*β* = 0.04, *p* > 0.05). This suggests that the effect of inclusive leadership on job performance may operate largely through grit and work engagement, though causal conclusions cannot be drawn given the cross‐sectional design. Further bootstrapped testing revealed that the total indirect effect of inclusive leadership on job performance via grit and work engagement was significant (*β* = 0.25, *p* < 0.001). Specifically, the indirect effect through grit was significant (*β* = 0.12, *p* < 0.001), as was the indirect effect through work engagement (*β* = 0.11, *p* < 0.001). The serial indirect effect of inclusive leadership on job performance via grit, followed by work engagement, was also significant (*β* = 0.02, *p* < 0.001). The full mediation model explained 27.4% of the variance in job performance. Regression coefficients and standard errors for each path are presented in Table S1. Notably, the three regression models within the mediation analysis showed no evidence of multicollinearity. Variance inflation factors (VIFs) for all variables ranged from 0.74 to 1.00, and tolerance values ranged from 1.00 to 1.36 (Table S1), well below the conventional thresholds, indicating problematic multicollinearity (VIF > 10 or tolerance < 0.1) [45].

**TABLE 3:** Total, direct, and indirect effects of the mediation models.

Effect (model 1: no control)	Effect size (β)	Bootstrap SE	Bootstrap 95% CI
Total effect (inclusive leadership ⟶ job performance)	0.29	0.05	[0.20, 0.38]
Direct effect (inclusive leadership ⟶ job performance)	0.04	0.05	[−0.05, 0.14]
Indirect effect	0.25	0.03	[0.19, 0.32]
Inclusive leadership ⟶ grit ⟶ job performance	0.12	0.02	[0.08, 0.17]
Inclusive leadership ⟶ work engagement ⟶ job performance	0.11	0.02	[0.06, 0.16]
Inclusive leadership ⟶ grit ⟶ work engagement ⟶ job performance	0.02	0.01	[0.01, 0.03]
Effect (Model 2: controlling for sociodemographic variables)			
Total effect (inclusive leadership ⟶ job performance)	0.29	0.05	[0.20, 0.38]
Direct effect (inclusive leadership ⟶ job performance)	0.04	0.05	[−0.05, 0.14]
Indirect effect	0.25	0.04	[0.18, 0.32]
Inclusive leadership ⟶ grit ⟶ job performance	0.12	0.02	[0.08, 0.17]
Inclusive leadership ⟶ work engagement ⟶ job performance	0.11	0.03	[0.06, 0.16]
Inclusive leadership ⟶ grit ⟶ work engagement ⟶ job performance	0.02	0.01	[0.01, 0.03]

*Note*: The sociodemographic variables controlled for in Model 2 were age, sex, years of education, income, and length of nursing work.

Abbreviations: CI, confidence interval; SE, standard error.

When sociodemographic factors (age, sex, educational years, income, and length of nursing work) were incorporated as covariates, the results of the mediation analysis remained largely consistent with the primary findings (see Table 3 and Figure 1b for details). In this adjusted model, the full mediation model explained 29.2% of the variance in job performance. Among the sociodemographic factors, job performance was significantly linked with income (*r* = 0.10, *p* = 0.038) and years of working experience (*r* = 0.10, *p* = 0.036) but not with age (*r* = 0.07, *p* = 0.134), sex (*r* = 0.02, *p* = 0.620), or educational years (*r* = 0.02, *p* = 0.736).

Next, to address potential common method bias, mediation analyses were reran with the first factor from the exploratory factor analysis included as a covariate. These analyses yielded results generally consistent with the primary findings. The indirect effect via grit (*β* = 0.05, 95% CI [0.02, 0.08], *p* < 0.05) and the indirect effect via work engagement (*β* = 0.05, 95% CI [0.02, 0.09], *p* < 0.05) remained significant. Crucially, the sequential indirect effect of inclusive leadership on job performance, mediated first by grit and subsequently by work engagement, also remained significant (*β* = 0.01, 95% CI [0.00, 0.03], *p* < 0.05).

Finally, to examine whether the inclusion of both secondary and tertiary hospitals introduced bias, we conducted moderated mediation analyses with hospital level (secondary vs. tertiary) as a moderator. The interaction terms for hospital level × inclusive leadership on grit (*β* = 0.03, *p* = 0.622), on work engagement (*β* = 0.01, *p* = 0.891), and the moderated indirect effects (all 95% CIs included zero) were nonsignificant, indicating that the mediation model operated similarly across hospital levels. These results support the appropriateness of combining both levels in the primary analysis.

## 4. Discussion

This study investigated the relationship between inclusive leadership and nurses’ job performance, specifically examining the individual and serial mediating roles of grit and work engagement. The results largely support our hypotheses, revealing a significant positive relationship between inclusive leadership and nurses’ job performance. Importantly, this relationship was statistically mediated by grit and work engagement, both individually and sequentially. These findings offer valuable insights into the psychological mechanisms through which inclusive leadership is linked to nurses’ job performance and carry significant implications for healthcare management and leadership development.

First, consistent with previous research [12–15], we found a significant positive total effect of inclusive leadership on nurses’ job performance (H1). However, our mediation analysis revealed that this relationship was statistically explained by grit and work engagement. Specifically, the direct effect of inclusive leadership on job performance became nonsignificant when these mediators were included, suggesting that inclusive leadership does not directly relate to job performance through simple command or directive influence. Instead, its association with job performance appears to operate through its ability to activate key intrapersonal motivational processes within nurses. When nurses perceive their leaders as open, accessible, and available, they are more likely to feel valued and supported [8, 46]. This, in turn, is associated with enhanced grit and work engagement, ultimately corresponding to better job performance. Therefore, our discussion will focus on these mediating mechanisms rather than a direct effect that was not supported in the full mediation model.

The finding that grit mediates the relationship between inclusive leadership and job performance (H2) is consistent with and extends prior research. For instance, Liu et al. found that grit directly predicted nurses’ job performance but did not examine organizational antecedents of grit [22]. Our study builds on this by identifying inclusive leadership as one such antecedent. Similarly, Schimschal et al. theoretically proposed that supportive leadership might be associated with grit but lacked empirical evidence [29]. Our findings offer initial empirical support for this proposition within a nursing context. The effect size of the indirect pathway via grit alone (*β* = 0.12) is comparable to that reported in similar mediation studies in nursing populations (e.g., Huang et al. reported indirect effects ranging from *β* = 0.09 to 0.15; [13]). Inclusive leadership, by fostering a supportive and empowering environment, may be associated with higher grit among nurses. When nurses perceive their leaders as valuing their contributions and supporting their professional development, they are more likely to embrace challenges, persist through setbacks, and maintain dedication to their work [20, 47]. This aligns with the JD–R theory [17], which posits that inclusive leadership functions as a crucial job resource, helping nurses manage work demands and achieve their goals. Nurses with higher grit may be better equipped to handle stressful situations, learn from their mistakes, and proactively pursue opportunities for professional development, ultimately corresponding to improved job performance [48, 49].

Similarly, the mediation of work engagement on the relationship between inclusive leadership and job performance (H3) emphasizes the importance of vigor, dedication, and absorption in nurses’ professional lives. This finding aligns with prior research revealing an association between inclusive leadership and higher work engagement among nurses in Egypt [31] and China [14]. It also complements numerous studies highlighting the positive association between nurses’ work engagement and job outcomes [50]. Our study extends these findings by demonstrating that work engagement mediates the link between inclusive leadership and job performance, going beyond simply showing a direct relationship between leadership and engagement or engagement and performance. Inclusive leadership, by fostering opportunities for autonomy, skill development, and social connection, is likely to be linked to nurses’ intrinsic motivation and willingness to invest effort in their work [51]. Engaged nurses tend to be more proactive, creative, and dedicated, which is associated with enhanced performance [25]. They experience a sense of energy, involvement, and efficacy that not only boosts productivity but also increases resilience to stress and reduces burnout risk [26, 27]. This finding aligns with self‐determination theory [18], which posits that individuals are more likely to be motivated and engaged when their basic psychological needs for autonomy, competence, and relatedness are met. This positive affective–cognitive engagement then corresponds to better performance.

The most novel finding of this study is the serial mediation effect of grit and work engagement (H4). Although statistically significant, the small indirect effect size (*β* = 0.02) warrants careful contextual interpretation, as recommended by the American Psychological Association [52]. Theoretically, this small serial effect is crucial because it reveals a sequential effect of grit and work engagement, beyond their parallel mediation. This supports the theoretical distinction between grit as a trait‐level resource and work engagement as a state‐level experience, implying that inclusive leadership may be associated with job performance through a cascading psychological process. Practically, the small effect size indicates that the sequential pathway is one of several mechanisms contributing to performance. The larger parallel indirect effects (grit alone: *β* = 0.12; work engagement alone: *β* = 0.11) highlight their independent contributions. However, even marginal improvements in performance, or an organization’s ability to predict it, can yield significant positive outcomes in high‐stakes settings [53]. Therefore, interventions aimed at boosting job performance through inclusive leadership should target both grit and work engagement simultaneously, rather than solely relying on a sequential chain reaction. This sequential model offers a more nuanced understanding of how leadership style may be associated with the day‐to‐day actions and outcomes of nursing staff [54]. Inclusive leaders create a supportive and empowering environment where nurses develop the passion and perseverance (grit) to pursue their long‐term goals. This increased grit, in turn, is associated with a stronger sense of dedication and absorption in their work, corresponding to higher levels of work engagement and improved job performance [33, 55]. This serial mediation effect highlights the interconnectedness of these psychological resources and underscores the importance of fostering both grit and work engagement to maximize the positive association between inclusive leadership and nurses’ job performance.

## 5. Limitations and Future Research Directions

While this study provides valuable insights into the relationship between inclusive leadership, grit, work engagement, and job performance among nurses, it is important to acknowledge its limitations. First, the cross‐sectional design, while informative for establishing correlations and testing statistical mediation, prohibits definitive causal inferences. Although our model is theoretically grounded, alternative explanations are possible. For instance, higher‐performing nurses might perceive their leaders as more inclusive, or engaged nurses might develop greater grit. Future research should employ longitudinal or experimental designs to establish causality and explore the dynamic relations between these variables over time. Methods such as cross‐lagged panel models or latent growth curve modeling could help determine whether inclusive leadership predicts subsequent changes in grit and work engagement, and whether these changes subsequently predict job performance over time.

Second, the data were collected using self‐report scales at a single time point, raising concerns about common method bias. Although Harman’s single‐factor test indicated that no single factor explained the majority of variance, and a statistical remedy was implemented, this test does not fully rule out common method variance. Specific concerns include social desirability bias (nurses may overreport positive perceptions of their leaders or their own performance) and consistency motif (respondents may attempt to maintain consistent responses across theoretically related constructs) [42]. Consequently, the positive correlations among study variables may be partially inflated by common method variance. To more rigorously address common method bias in future research, we recommend employing multisource data collection strategies, such as obtaining supervisor ratings of job performance [56] or peer assessments of work engagement, as well as utilizing temporal separation (e.g., multiwave designs with time‐lagged measurements). Moreover, the study used a mix of 5‐point and 7‐point Likert scales for different measures. Although we mitigated potential distortions through data standardization and attention checks, mixing scale anchors may still contribute to respondent fatigue or measurement artifacts. Methodological research suggests that mixing scale anchors, when properly managed, may actually reduce common method variance by disrupting consistency motifs [42]; nevertheless, future studies should consider using a uniform response scale (e.g., all 7‐point) to simplify interpretation and reduce cognitive load.

Third, the study sample was recruited using convenience sampling from one province in China. Although Sichuan Province offers diversity in healthcare settings, the findings may not fully generalize to other regions with different cultural norms, economic conditions, or healthcare policies. The overwhelming majority of participants were female (93.7%). While this reflects the gender distribution in nursing globally [57], it may limit generalizability. Perceptions of inclusive leadership could differ by gender; research suggests that female nurses may have different expectations and sensitivities regarding leader accessibility compared to male nurses [58]. Additionally, within the Chinese cultural context, characterized by relatively high power distance, the concept of inclusive leadership may be interpreted differently than in more egalitarian cultures [59]. Chinese nurses might perceive leader accessibility through hierarchical norms that emphasize deference to authority. Therefore, the current findings may not generalize to more gender‐balanced nursing populations or to countries with different cultural norms regarding leadership and hierarchy. Future research should examine these relationships in diverse cultural and gender contexts.

Fourth, unmeasured contextual factors may influence the observed relationships. For instance, team climate (e.g., cohesion and psychological safety), workload demands (e.g., nurse‐to‐patient ratios), and organizational culture (e.g., support for professional development) could moderate or confound the relationships among inclusive leadership, grit, work engagement, and job performance [60]. The JD–R model, which guided our theoretical framework, explicitly recognizes the interaction between job demands and resources in influencing employee outcomes [17]. However, we did not directly measure certain job demands (e.g., time pressure) or job resources (e.g., social support). Future research should incorporate these contextual variables to more fully test the current mediation model within the JD–R framework. Furthermore, exploring the influence of specific inclusive leadership behaviors (e.g., promoting diversity and empowering team members; [61]) on grit and work engagement could also provide a more granular understanding of the underlying processes. Besides, the study included both secondary and tertiary hospitals, which differ in resources, patient acuity, and workload demands. Although our sensitivity analyses revealed no statistically significant differences in the mediation model across hospital levels, unmeasured contextual factors may still confound the observed relationships. Future research should systematically record hospital level and other organizational characteristics as potential moderators to formally test contextual influences.

## 6. Implications for Nursing Management and Practice

The findings of the present study offer several practical implications for healthcare management and leadership development. First, targeting the inclusive leadership ⟶ grit pathway: Healthcare organizations should train nurse managers in specific behaviors that foster grit among staff [62]. These include (a) providing perseverance‐focused feedback that acknowledges effort and improvement rather than solely praising outcomes; (b) sharing stories of how senior nurses overcame challenges and persisted through difficult cases; (c) reframing setbacks (e.g., patient complications and medical errors) as learning opportunities rather than failures; and (d) encouraging nurses to set long‐term professional development goals (e.g., specialty certifications and advanced degrees) with structured milestones.

Second, targeting the inclusive leadership ⟶ work engagement pathway: Nurse managers can enhance work engagement by (a) conducting weekly conversations that solicit input on workflow improvements; (b) maintaining visible and predictable office hours when staff can raise concerns (demonstrating accessibility); (c) responding to staff emails and messages within 24 h (demonstrating availability); and (d) implementing shared governance models where staff nurses participate in unit‐level decision‐making (e.g., scheduling and protocol selection). These behaviors directly address the three dimensions of work engagement: vigor (energy), dedication (involvement), and absorption (focus) [63].

Third, targeting the sequential grit ⟶ work engagement pathway: Even when nurses develop grit, organizations need systems to translate that perseverance into daily engagement. Specific strategies include (a) providing protected time for grit‐oriented nurses to pursue quality improvement projects; (b) recognizing and rewarding sustained effort; and (c) creating career ladder programs that provide clear pathways from gritty perseverance to tangible professional advancement. Mentorship programs pairing gritty senior nurses with less experienced staff can also help transmit both perseverance and engagement simultaneously.

Finally, healthcare organizations should implement multicomponent interventions that address all three pathways simultaneously. For example, a leadership development program might include (a) a workshop for nurse managers on inclusive leadership behaviors (openness, accessibility, and availability; [64]); (b) monthly “grit rounds” where managers discuss strategies for fostering staff perseverance and passion; and (c) quarterly engagement surveys with action planning based on results. Such comprehensive approaches are more likely to produce meaningful improvements in nurse job performance than single‐focus interventions.

## 7. Conclusion

In conclusion, this study provides fresh evidence that inclusive leadership is positively associated with nurses’ job performance. Furthermore, our findings, within a cross‐sectional sample, demonstrate that this relationship is statistically mediated by grit and work engagement, acting both individually and sequentially. These findings underscore the importance of fostering inclusive leadership practices and promoting grit and work engagement among nurses to enhance their job performance, which may, according to prior research, contribute to improved patient outcomes. By investing in leadership development and cultivating these psychological resources, healthcare organizations can create a more supportive, engaging, and productive work environment for nurses, thereby fostering a more sustainable and effective healthcare system.

## Funding

This work was supported by the Key Research and Development Program of Sichuan Province (Grant No. 2023YFS0084).

## Ethics Statement

Ethical approval for this research was obtained from the Ethics Committee of West China Hospital of Sichuan University (approval number: 2021–1216). Written informed consent was secured from each participant, signifying their comprehension of the study’s protocols and their voluntary and anonymous engagement.

## Conflicts of Interest

The authors declare no conflicts of interest.

## Supporting Information

Additional supporting information can be found online in the Supporting Information section.

## Supporting information


**Supporting Information** Table S1. Regression analysis of the relationship between variables.

## Data Availability

The data that support the findings of this study are available from the corresponding author upon reasonable request.
