# Supplementary material for: The Association Between Inclusive Leadership and Job Performance in Nurses: Exploring the Mediation Roles of Grit and Work Engagement Based on a Cross‐Sectional Study
Source: J Nurs Manag. 2026 Jun 30;2026:9399937. doi: 10.1155/jonm/9399937 (PMC13315811; doi:10.1155/jonm/9399937)
Supplement: Supplementary file 1 — Supporting Information Table S1. Regression analysis of the relationship between variables. [file JONM-2026-9399937-s001.docx]

**Supplementary Information for**

***‘The association between inclusive leadership and job performance in nurses: Exploring the mediation roles of grit and work engagement based on a cross-sectional study’***

**Table S1.** Regression analysis of the relationship between variables

**Table S1.** Regression analysis of the relationship between variables

| **Model** | **Outcome variable** | **Predictor variable** | **B** | **SE** | **β** | **t** | **VIF** | **Tolerance** |
| --- | --- | --- | --- | --- | --- | --- | --- | --- |
| Model 1 | Grit | Inclusive leadership | 0.27 | 0.03 | 0.36 | 7.97^***^ | 1.00 | 1.00 |
| Model 2 | Work engagement | Inclusive leadership | 1.32 | 0.15 | 0.39 | 8.81^***^ | 0.87 | 1.15 |
|  |  | Grit | 0.96 | 0.20 | 0.21 | 4.75^***^ | 0.87 | 1.15 |
| Model 3 | Job performance | Inclusive leadership | 0.03 | 0.04 | 0.04 | 0.87 | 0.74 | 1.36 |
|  |  | Grit | 0.34 | 0.05 | 0.34 | 7.46^***^ | 0.83 | 1.21 |
|  |  | Work engagement | 0.06 | 0.01 | 0.27 | 5.59^***^ | 0.74 | 1.35 |

Note. SE, standard error; VIF, variance inflation factor; ^***^, p < 0.001.
